# Ecological co-burden patterns of GERD and asthma: a multi-method analysis of representative global regions

**DOI:** 10.3389/fnut.2026.1774196

**Published:** 2026-03-26

**Authors:** Yinghan Deng, Zhaoliang Xie, Lixue You, Jing Wang, Meijuan Wu

**Affiliations:** 1 Department of Respiratory and Critical Care Medicine, Sanming Second Hospital, Sanming, Fujian, China; 2 Department of Pulmonary and Critical Care Medicine, The Second Affiliated Hospital of Fujian Medical University, Quanzhou, Fujian, China

**Keywords:** asthma, comorbidity, epidemiologic transition, gastroesophageal reflux disease, global burden of disease, risk factors

## Abstract

**Background:**

Gastroesophageal reflux disease (GERD) and asthma often co-occur, yet their joint trajectories and shared drivers may differ across regions.

**Methods:**

Using Global Burden of Disease (GBD) 2023 estimates (1994–2023), we compared East Asia, Tropical Latin America, and High-income North America. Age-specific associations were assessed by Spearman correlation. Trends were quantified with Joinpoint regression (AAPC) and forecasts (2024–2033) generated using ETS/ARIMA. Shared drivers were screened using Random Forest with SHAP. Associations between exposures and disease burden were quantified using negative binomial regression of prevalence counts with a log(population) offset, and geographic heterogeneity tested using exposure × region interaction models. Granger causality and multigroup structural equation modelling (SEM) were used to explore temporal directionality and pathways.

**Results:**

In 2023, GERD age-standardised prevalence was highest in Tropical Latin America (16,591 per 100,000) and lowest in East Asia (4,465 per 100,000), yet East Asia carried 84.6 million GERD cases. Trends showed increasing GERD and asthma in East Asia, rising asthma in Tropical Latin America, and declining GERD but increasing asthma in High-income North America. In pooled models, asthma was positively associated with diet high in red meat (RR 1.66, 95% CI 1.51–1.84), diet low in vegetables (1.56, 1.11–2.20), high fasting plasma glucose (1.13, 1.08–1.19), low physical activity (1.16, 1.02–1.33), and suboptimal breastfeeding (1.36, 1.23–1.52). GERD was positively associated with sugar-sweetened beverages (1.51, 1.25–1.82), suboptimal breastfeeding (1.34, 1.27–1.42), and high fasting plasma glucose (1.03, 1.00–1.06). Interaction models indicated strong regional effect modification, with fasting glucose positively associated with asthma in East Asia (1.23, 1.18–1.28) and Tropical Latin America (1.16, 1.14–1.19) but inversely in High-income North America (0.97, 0.95–0.99).

**Conclusion:**

GERD–asthma comorbidity follows distinct regional trajectories, and shared metabolic/lifestyle drivers show marked geographic heterogeneity, supporting context-specific prevention strategies.

## 1 Introduction

Gastroesophageal reflux disease (GERD) and asthma are two of the most prevalent non-communicable chronic diseases worldwide, imposing a substantial burden on public health systems and reducing patients’ quality of life (1, 2). According to previous studies utilising the GBD database, an estimated 260 million individuals had asthma globally in 2021, with the highest age-standardised prevalence rate (ASPR) observed in high-income North America (3). Additionally, there were 783.95 million cases of gastroesophageal reflux disease (GERD) globally in 2019, with Tropical Latin America exhibiting the highest ASPR (4, 5). Previous studies have suggested a potential association between these two conditions (6, 7), and have also indicated a high prevalence of GERD among asthma patients (8–10). Traditionally, this comorbidity has largely been attributed to direct causal pathways: the “reflux theory” (where micro-aspiration of gastric acid induces bronchoconstriction) (11–13) and the “reflex theory” (involving vagal nerve stimulation) (12, 14). However, this simple “causal relationship” paradigm may insufficiently explain the concurrent rise of both diseases in rapidly developing regions. An emerging “common soil” hypothesis proposes that GERD and asthma may share upstream risk factors, such as metabolic syndrome and obesity (15–19), which simultaneously inflict damage on both the digestive and respiratory systems. Despite this biological plausibility, existing research is predominantly limited to cross-sectional clinical cohorts or single-nation analyses, lacking a longitudinal, global perspective to clarify the complex interactions between environmental exposures, metabolic transitions, and disease trajectories.

Given that the epidemiological transition is not uniform across countries and regions, risk factors of GERD–asthma comorbidity (eg, metabolic risks, diet, and environmental exposures) may differ in direction and magnitude across settings (20). Disease-specific GBD analyses have shown substantial geographic variation in the burden of asthma and GERD across countries and regions (3–5), while the broader GBD 2023 capstone analyses also document marked cross-national and subnational heterogeneity in diseases, injuries, mortality, and risk-attributable burden (21–23), We therefore hypothesised that overlooking such heterogeneity could lead to overly generalised and less effective public health strategies. Accordingly, the present analysis explicitly quantified region-specific trajectories and effect modification.

## 2 Methods

Based on clinical and epidemiological evidence that GERD can precede or exacerbate asthma through reflux-related mechanisms (micro-aspiration and vagally mediated reflex bronchoconstriction) (6, 8, 14, 24). we treated GERD as a potential upstream condition in analyses while also allowing for shared upstream risk factors such as metabolic dysfunction and lifestyle factors (15–19). We conducted a comparative, region-level analysis using aggregated GBD 2023 estimates across three SDI-stratified regions with contrasting GERD burden profiles (East Asia, Tropical Latin America, High-income North America; selection quantified in Supplementary Tables S1, S2). The present study used publicly available estimates from the Global Burden of Disease Study 2023, and the overall GBD 2023 study design, data processing pipeline, and core modelling strategies have been described in the main demographic, causes-of-death, and diseases/injuries/risk-factor capstone publications (21–23).

In the GBD framework, asthma prevalence is modelled using a reference case definition of physician-diagnosed asthma plus wheezing during the previous 12 months, with ICD-9 code 493 and ICD-10 codes J45-J46, alternative input definitions are cross-walked to this reference definition during model harmonisation (3, 21–23). GERD prevalence is modelled as symptomatic gastro-oesophageal reflux disease based on typical reflux symptoms, specifically heartburn and/or acid regurgitation, as synthesised from population-based studies and adjusted for between-study design differences within the GBD non-fatal modelling framework (5, 21–23).

### 2.1 Correlation and trend analysis

We extracted age-standardised prevalence rates and prevalence counts for gastroesophageal reflux disease (GERD) and asthma from the GBD 2023 Results Tool^1^ for East Asia, Tropical Latin America, and High-income North America from 1994 to 2023. All estimates were analysed as reported by GBD 2023 and interpreted according to the reference case definitions described above. Age-specific associations between GERD and asthma were assessed using Spearman correlation coefficients (*ρ*) across years within each age group and region. Temporal trends in age-standardised prevalence were quantified using Joinpoint regression, reporting annual percentage change and average annual percentage change (AAPC) with 95% confidence intervals using Monte Carlo permutation tests (25). For forecasting (2024–2033), we prioritised ETS exponential smoothing models for non-stationary annual series and compared candidate models using information criteria and residual diagnostics; if ETS failed to converge or performed poorly, we switched to auto-selected ARIMA models, and, if needed, Holt’s linear method (26). Model performance was checked by out-of-sample validation on the most recent years where feasible (MAPE/RMSE).

### 2.2 Risk factor analysis

To investigate shared upstream risk factors beyond disease-specific comparative risk assessment outputs, we applied a two-stage, data-driven screening strategy. First, we compiled the most detailed GBD risk exposures with available annual summary exposure values (SEV) across the study period; from an initial catalogue of 68 detailed risks, 24 exposures with complete annual coverage and plausible relevance to both GERD and asthma were retained for modelling: high body-mass index, high fasting plasma glucose, smoking, secondhand smoke, alcohol use, low physical activity, ambient particulate matter pollution, ambient ozone pollution, household air pollution from solid fuels, nitrogen dioxide pollution, occupational asthmagens, occupational particulate matter/gases/fumes, occupational exposure to formaldehyde, occupational exposure to sulphuric acid, high temperature, low temperature, diet low in fruits, diet low in vegetables, diet low in fibre, diet high in red meat, diet high in processed meat, diet high in sugar-sweetened beverages, diet high in sodium, and suboptimal breastfeeding. Second, we fitted Random Forest models (27) with leave-one-out cross-validation (LOOCV) to reduce overfitting and used SHapley Additive exPlanations (SHAP) to quantify global feature importance (28). Candidate positive risk factors were confirmed when the Spearman correlation between each feature’s value and its SHAP contribution was >0.15, indicating a consistent positive direction in the non-linear model. This framework has been used in recent GBD-based co-occurrence research to identify shared drivers under a new analytic approach (29). The *ρ* > 0.15 threshold was pre-specified to indicate at least a small-to-moderate monotonic association between feature value and SHAP contribution, prioritising directional stability over sensitivity in small samples.

### 2.3 Risk quantification and heterogeneity analysis

To quantify exposure–disease associations while considering the count-data structure and overdispersion, we used negative binomial regression models with a log link (30). For pooled analyses, the dependent variable was prevalence count (number of cases; all ages; both sexes) and we included an offset term for the log population, yielding prevalence rate ratios (PRRs). Exposures were z-standardised; therefore PRRs correspond to a 1 SD increase in SEV. Models adjusted for region (fixed effects) and calendar year. Robust (HC0) standard errors were used; given only three regions, cluster-robust standard errors were treated as a sensitivity analysis. Regional heterogeneity was formally tested using exposure×region interaction models (likelihood-ratio tests), and region-specific PRRs were derived from the fitted interaction terms. When multivariable models did not converge within a region due to limited longitudinal variance of certain SEVs, we reported univariable estimates or excluded unstable estimates (pre-specified) to avoid misleading inference.

### 2.4 Causal inference and path analysis

To explore the temporal and mechanistic relationships among GERD, asthma, and related drivers, we conducted two advanced analyses.

Granger Causality Test: This test was performed on the time-series data from each region to determine the temporal precedence (predictive causal relationship) between the prevalence rates of GERD and asthma.

Structural Equation Modelling (SEM): This model assessed the direct effects of relevant risk factors on both GERD and asthma, as well as the indirect effects mediated through GERD. Multigroup SEM was employed to compare the path coefficients across the three regions, evaluating the geographic heterogeneity of the underlying pathogenic mechanisms.

All statistical tests were two-sided, and a *p*-value < 0.05 was considered statistically significant. All statistical analyses were performed using R software (version 4.3.1).

## 3 Results

### 3.1 Correlation analysis, spatiotemporal trends and future predictions

**Figure 1:**
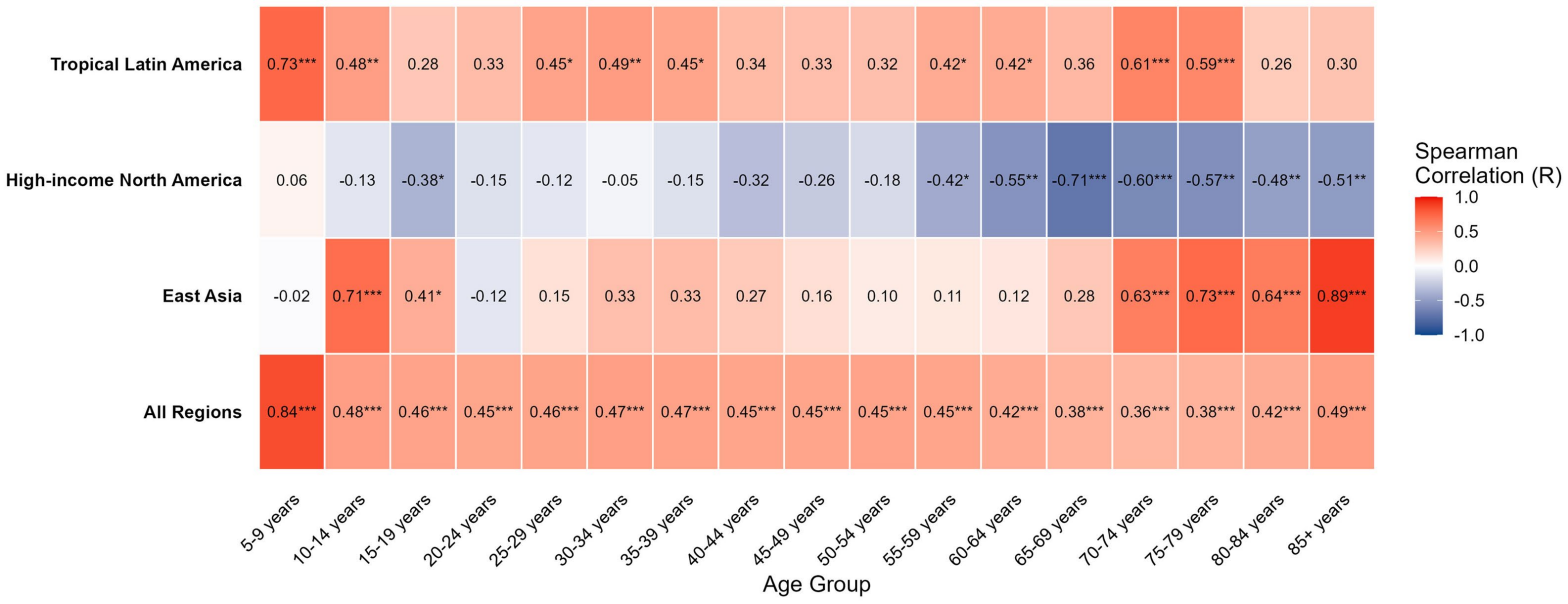
Analysis of the correlation between the prevalence of gastroesophageal reflux disease (GERD) and asthma across different age groups. ^*^p < 0.05; ^**^p < 0.01;^***^p < 0.001.

Correlation analysis uncovered significant heterogeneity in the GERD-asthma relationship across different age groups. In East Asia, the correlation was weak during young and middle adulthood but increased sharply among the elderly population (>70 years), displaying a characteristic “geriatric comorbidity” profile. In contrast, North America exhibited an anomalous negative disease correlation across almost all age groups, which was particularly pronounced among the elderly (>65 years). In Tropical Latin America, a significant positive correlation was observed in children and adolescents (5–14 years), young adults (25–39 years), and the elderly (70–79 years) (Figure 1).

**Figure 2:**
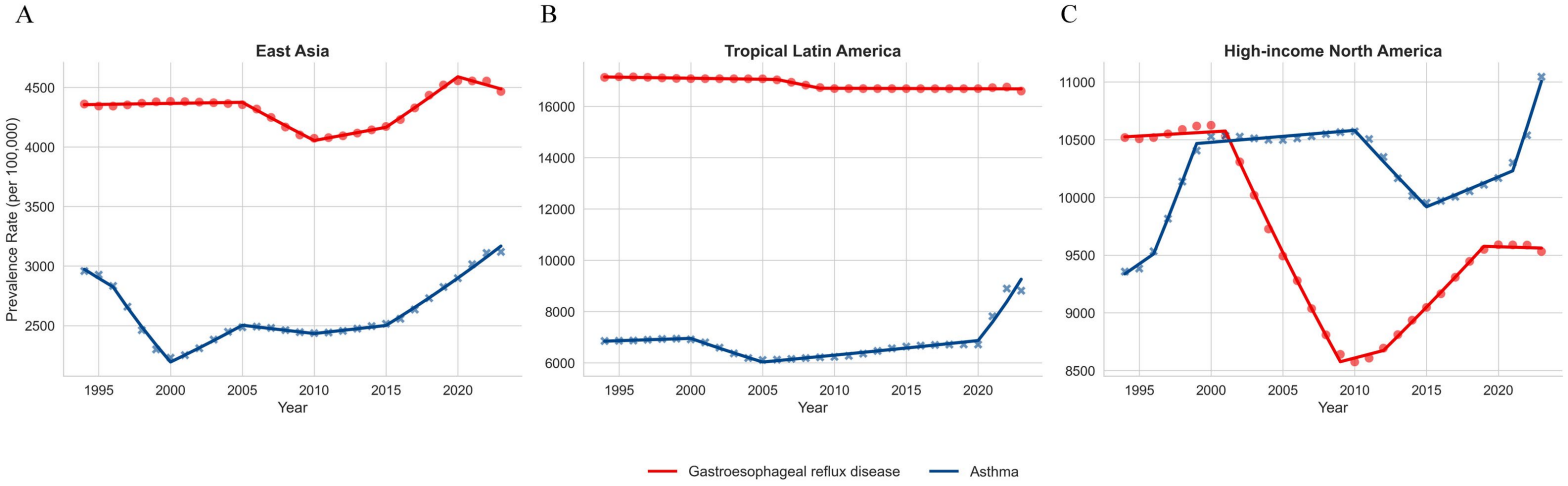
**(A)** Trends in age-standardized prevalence of asthma and gastroesophageal reflux disease (GERD) in East Asia, 1992–2023. **(B)** Trends in age-standardized prevalence of asthma and gastroesophageal reflux disease (GERD) in Tropical Latin America, 1992–2023. **(C)** Trends in age-standardized prevalence of asthma and gastroesophageal reflux disease (GERD) in High-income North America, 1992–2023.

Between 1994 and 2023, the age-standardised rates (ASR) of GERD and asthma exhibited distinctly divergent trajectories across the different regions. Joinpoint regression analysis revealed a synchronous upward trend in East Asia, with both GERD (AAPC = 0.10) and asthma (AAPC = 0.22) showing consistent growth. In Tropical Latin America, the asthma burden increased at a faster rate (AAPC = 1.05). Conversely, High-income North America presented a divergent pattern: while asthma prevalence continued to rise (AAPC = 0.57), GERD prevalence showed a significant declining trend (AAPC = −0.33) (Figure 2; Supplementary Table S3).

**Figure 3:**
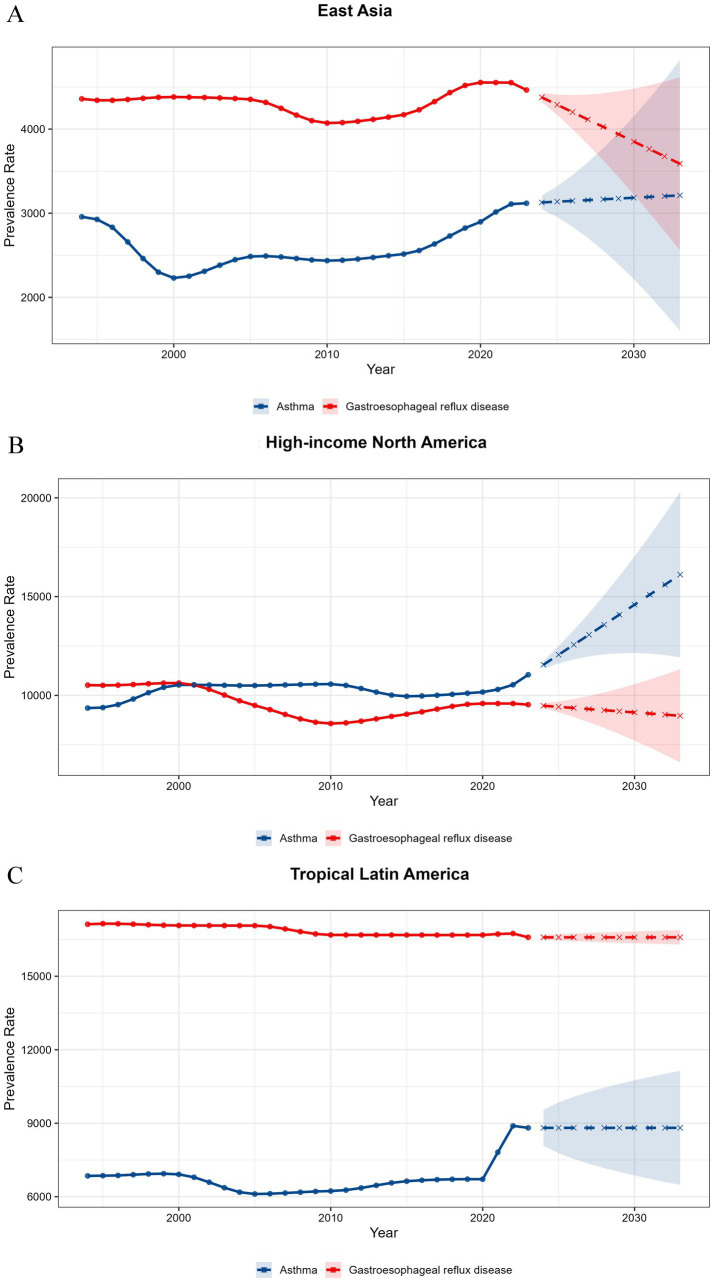
**(A)** Forecasted trends in age-standardized prevalence of asthma and gastroesophageal reflux disease (GERD) in East Asia, 2024–2033. **(B)** Forecasted trends in age-standardized prevalence of asthma and gastroesophageal reflux disease (GERD) in High-income North America, 2024–2033. **(C)** Forecasted trends in age-standardized prevalence of asthma and gastroesophageal reflux disease (GERD) in Tropical Latin America, 2024–2033.

Time-series forecasting based on ETS/ARIMA models projected that over the next decade, GERD prevalence in East Asia is expected to decline continuously, whereas asthma prevalence is predicted to further increase. In North America, asthma prevalence has been higher than GERD for most of the past three decades, and this divergence is forecasted to widen. In Tropical Latin America, the prevalence of GERD has remained substantially higher than that of asthma, a trend predicted to remain relatively stable in the coming decade (Figure 3; Supplementary Table S5).

### 3.2 Machine learning-based driver screening

**Figure 4:**
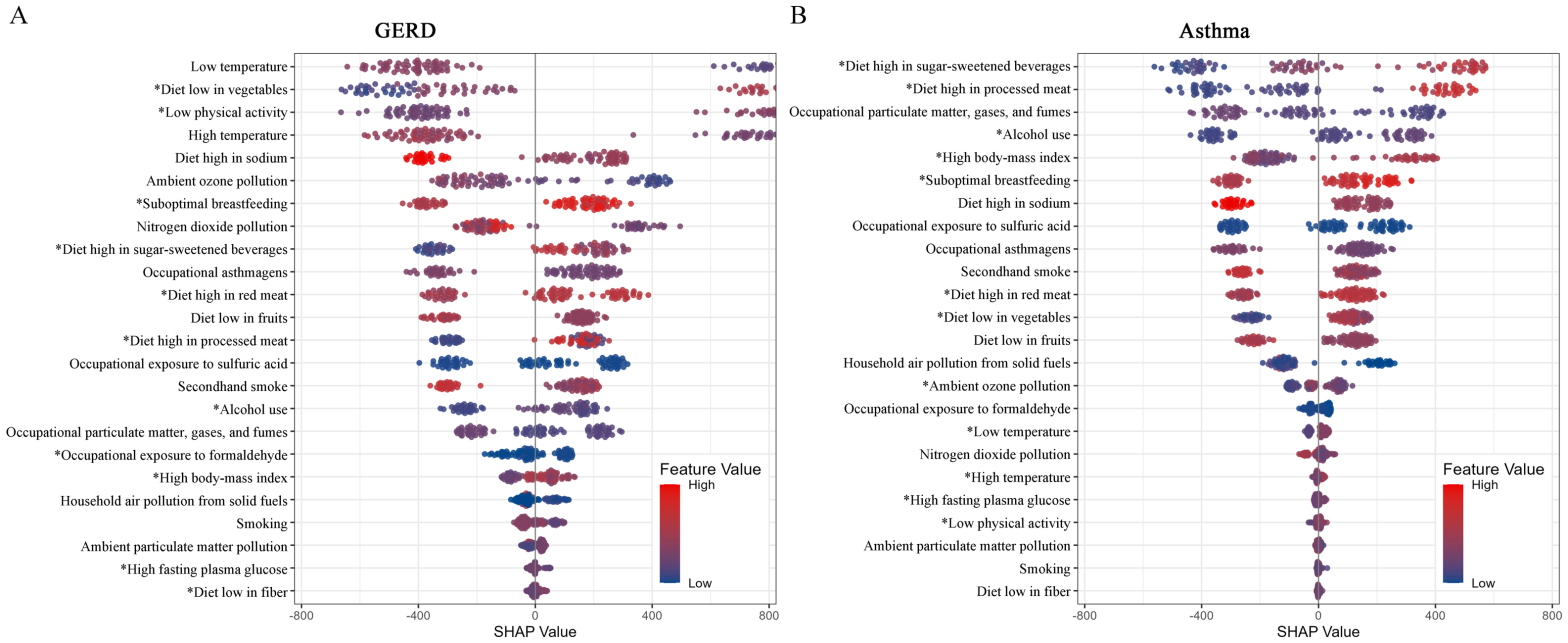
SHAP analysis and negative binomial regression analysis of risk factors associated with the prevalence of gastroesophageal reflux disease (GERD) and asthma. **(A)** SHAP analysis of risk factors associated with GERD prevalence; **(B)** SHAP analysis of risk factors associated with asthma prevalence. *represents significant positive rick factors.

In the pooled Random Forest model, metabolic and dietary factors were identified as primary predictors of both GERD and asthma prevalence. SHAP analysis pinpointed High fasting plasma glucose, High body-mass index, Alcohol use, Diet low in vegetables, Diet high in red meat, Diet high in processed meat, Diet high in sugar-sweetened beverages, and Low physical activity as common positive drivers for both diseases (Figure 4).

### 3.3 Risk quantification and regional heterogeneity

**Figure 5:**
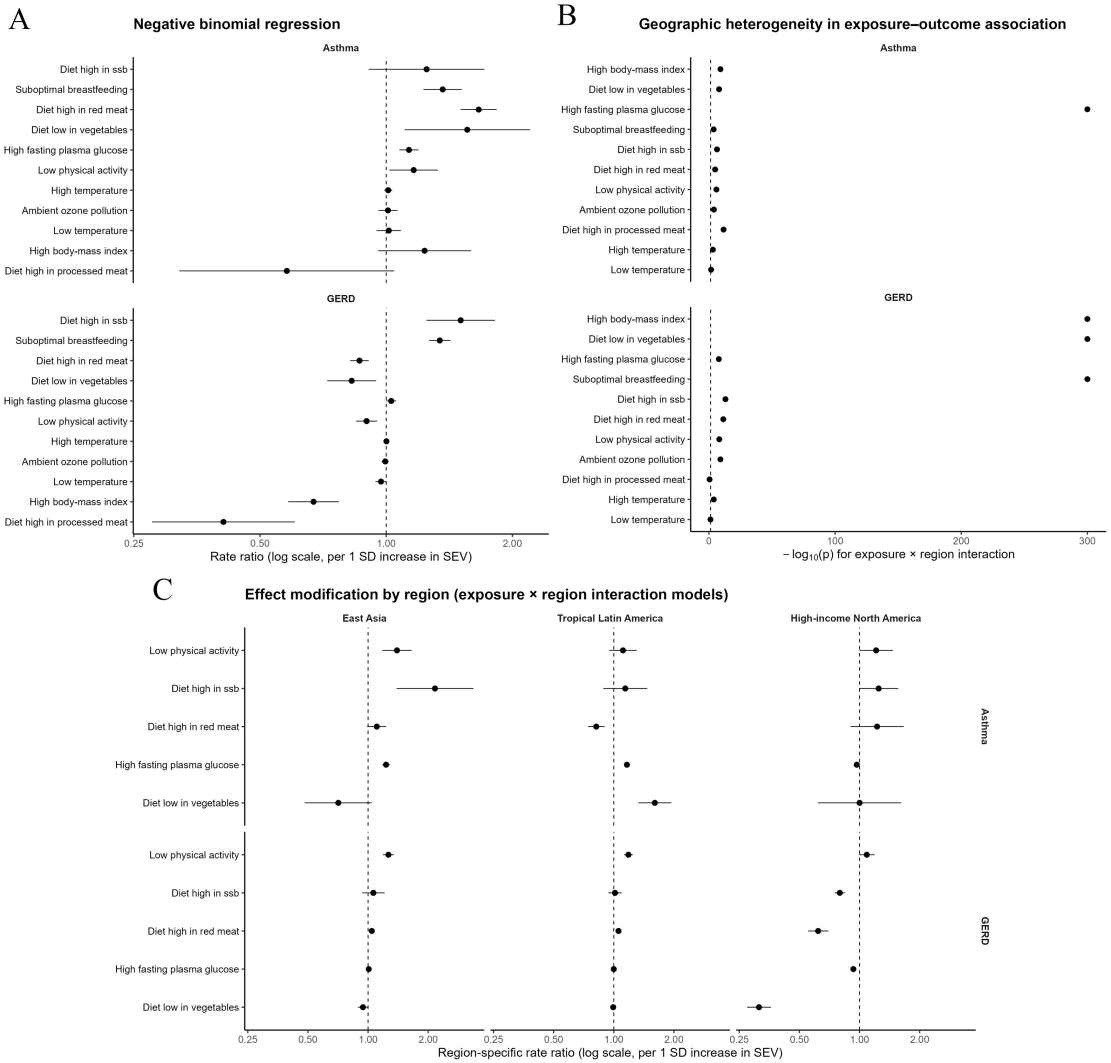
Further univariable negative binomial regression analysis of risk factors for gastroesophageal reflux disease (GERD) and asthma prevalence in three regions. **(A)** Multivariable negative binomial regression analysis of risk factors for asthma and gastroesophageal reflux disease (GERD) prevalence in three regions; **(B)** Analysis of geographic heterogeneity in exposure-outcome associations of asthma and gastroesophageal reflux disease (GERD); **(C)** Analysis of interaction between exposure factors and region in asthma and gastroesophageal reflux disease (GERD).

In the pooled negative binomial regression models (prevalence counts with a log(population) offset), several SHAP-selected exposures were independently associated with disease burden. For asthma, suboptimal breastfeeding (RR 1.36, 95%CI:1.23–1.52), high fasting plasma glucose (RR 1.13, 95%CI:1.08–1.19), diet low in vegetables (RR 1.56, 95%CI:1.11–2.20), diet high in red meat (PRR 1.66, 95%CI:1.51–1.84), and low physical activity (RR 1.16, 95%CI:1.02–1.33) were associated with higher prevalence (Figure 5). For GERD, diet high in sugar-sweetened beverages (RR 1.51, 95%CI:1.25–1.82), suboptimal breastfeeding (PRR 1.34, 95%CI:1.27–1.42), and high fasting plasma glucose (RR 1.03, 95%CI:1.00–1.06) showed positive associations, whereas several exposures exhibited inverse associations in the pooled model (e.g., high body-mass index RR 0.67, 95%CI:0.58–0.77). To formally assess geographic heterogeneity, exposure × region interaction models demonstrated significant effect modification for most exposures (likelihood ratio test *p* < 0.05). Notably, fasting plasma glucose was positively associated with asthma in East Asia (RR 1.23, 95%CI:1.18–1.28) and Tropical Latin America (RR 1.16, 95%CI:1.14–1.19), but inversely associated in High-income North America (RR 0.97, 95%CI:0.95–0.99). For GERD, the glucose association was near null in East Asia and Tropical Latin America, but inverse in High-income North America (RR 0.93, 95%CI:0.92–0.94) (Figure 5; Supplementary Tables S6, S7).

### 3.4 Causal inference and pathogenic mechanism analysis

To clarify the temporal and mechanistic links, we integrated findings from the Granger causality test and Structural Equation Modelling (SEM).

**Figure 6:**
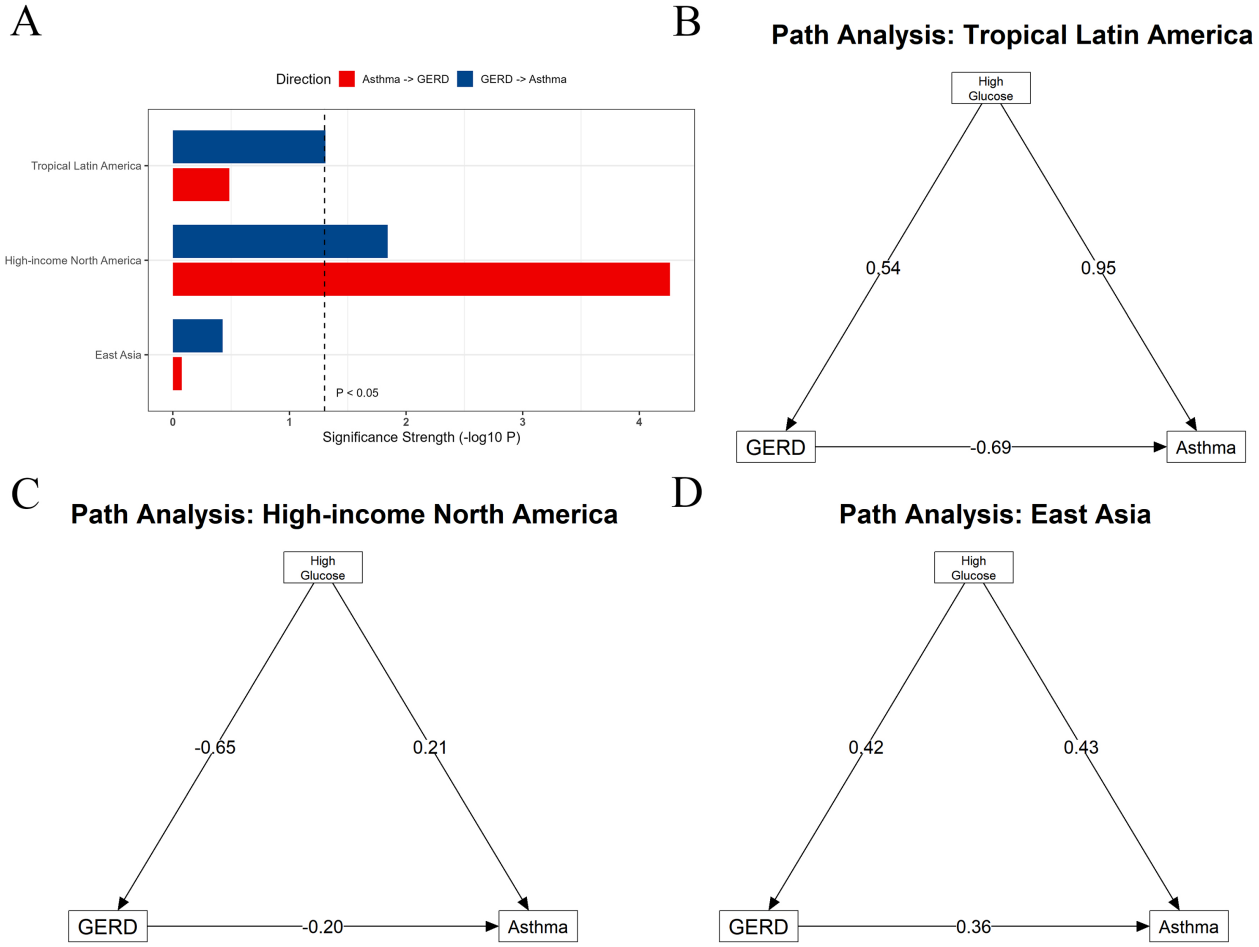
Granger causality test and structural equation modelling (SEM). **(A)** Time-lagged analysis (Granger causality test) of the prevalence of gastroesophageal reflux disease (GERD) and asthma; **(B)** Structural equation modelling (SEM) analysis of gastroesophageal reflux disease (GERD), asthma, and high fasting plasma glucose in tropical Latin America; **(C)** Structural equation modelling (SEM) analysis of gastroesophageal reflux disease (GERD), asthma, and high fasting plasma glucose in high-income North America; **(D)** Structural equation modelling (SEM) analysis of gastroesophageal reflux disease (GERD), asthma, and high fasting plasma glucose in East Asia.

The Granger test revealed a significant unidirectional causal prediction from GERD to asthma in Tropical Latin America (*p* = 0.049). In North America, a significant bidirectional causal relationship was observed. Notably, no significant time-lagged relationship was found in East Asia (Figure 6A). Building on the machine learning screening (Random Forest/SHAP) and the interaction NBR results demonstrating pronounced region-specific associations for high fasting plasma glucose, we incorporated high fasting plasma glucose into the subsequent multigroup SEM analysis (Figure 6).

Multigroup SEM further elucidated distinct pathogenic pathways. In East Asia, High fasting plasma glucose demonstrated significant and concurrent direct effects on both GERD (Std. β = 0.42) and asthma (Std. β = 0.43), and GERD also had a significant direct effect on asthma (Std. β = 0.360) (Figure 6D). In Tropical Latin America, High fasting plasma glucose showed even more pronounced direct effects on GERD (Std. β = 0.54) and asthma (Std. β = 0.95), but the effect of GERD on asthma was negative (Std. β = −0.69) (Figure 6B). Conversely, in High-income North America, the direct effects of High fasting plasma glucose on both GERD and asthma were markedly attenuated, even showing a negative effect on GERD (Std. β = −0.65) (Figure 6C).

## 4 Discussion

This study conducted a multidimensional analysis of the GERD-asthma comorbidity burden across three regions representing distinct Socio-demographic Index (SDI) levels. While the pooled population analysis confirmed a positive correlation between the two diseases, the most significant finding lies in the profound divergence of epidemiological patterns. Over the past three decades, East Asia (middle-to-high SDI) exhibited a simultaneous rapid increase in GERD and asthma burdens. Tropical Latin America (middle SDI), identified as a global GERD “hotspot region” with the highest prevalence, maintained a stable, high prevalence rate over this period, while its asthma prevalence showed a marked increase post-2020 (APC 2020–2023: 10.46) (Supplementary Table S4). Conversely, High-income North America (high SDI) presented a unique declining trend in GERD burden, alongside a notable rise in asthma burden in recent years. This tripartite divergence indicates that although the GERD-asthma comorbidity is widespread, the manifestations and potential drivers of this comorbidity are strongly shaped by regional context (e.g., demographic structure, lifestyle exposures, and health system accessibility).

To deconstruct the shared aetiology of this comorbidity, we triangulated evidence from nonlinear machine learning (Random Forest with SHAP) and count-based negative binomial regression (NBR) using prevalent cases with a log(population) offset. SHAP consistently highlighted metabolic dysfunction (high fasting plasma glucose, high body-mass index, and low physical activity) and westernised dietary patterns (e.g., high red meat intake, low vegetable intake, and high sugar-sweetened beverage consumption) as key contributors. In pooled multivariable NBR, asthma was positively associated with high fasting plasma glucose (RR:1.13, 95% CI:1.08–1.19), low physical activity (1.16, 1.02–1.33), high red meat intake (1.66, 1.51–1.84), low vegetable intake (1.56, 1.11–2.20), and suboptimal breastfeeding (1.36, 1.23–1.52). For GERD, positive associations were observed for high fasting plasma glucose (1.03, 1.00–1.06), high sugar-sweetened beverage intake (1.51, 1.25–1.82), and suboptimal breastfeeding (1.34, 1.27–1.42). These findings are consistent with the “common soil” hypothesis in which upstream metabolic and lifestyle exposures jointly shape digestive and respiratory morbidity (31–34). Importantly, formal exposure-by-region interaction models demonstrated substantial effect modification for most exposures, indicating that both the magnitude and direction of associations can differ across epidemiological transition contexts and health system environments (20).

Although East Asia currently has the lowest GERD age-standardised prevalence rate (ASPR) among the three regions, its enormous population base results in an extremely high absolute case burden (35). Our trend analysis revealed a “synchronous surge” pattern, characterised by parallel increases in the prevalence of both GERD and asthma (with positive AAPCs) and an absence of a time lag. In interaction NBR models, a 1-SD increase in fasting plasma glucose was associated with higher asthma rates in East Asia (RR approximately 1.23) but only a near-null association with GERD (RR approximately 1.01), suggesting that metabolic dysregulation may contribute more strongly to the respiratory burden in this ageing population. The SEM analysis indicated that High fasting plasma glucose drives both diseases with comparable strength, suggesting that the comorbidity pattern in East Asia is primarily driven by synchronous escalation of both disease burdens fueled by related metabolic abnormalities. Furthermore, our age-correlation analysis found that this comorbidity is weaker in younger adults but intensifies sharply among the elderly population (>70 years). Therefore, we attribute this regional pattern to the synergistic effect of rapid metabolic transition and population ageing. In East Asia, the interplay of declining physiological function associated with ageing and cumulative metabolic exposure (36) fosters a distinct form of “geriatric comorbidity.” This also partly explains the previously observed phenomenon in GERD burden studies for East Asia: a low age-standardised prevalence but a high absolute burden.

Tropical Latin America exhibits the highest GERD age-standardised prevalence rate (ASPR) and the fastest growth rate in asthma prevalence (AAPC = 1.05) among the three regions. The SEM analysis revealed significant path coefficients for High fasting plasma glucose on both GERD and asthma in this region. Furthermore, the lag analysis indicated a unidirectional temporal relationship (from GERD to asthma), which is consistent with the classic reflux-induced hypothesis in which reflux may contribute to respiratory symptoms through micro-aspiration and neurogenic reflex pathways (37). Taken together, in this middle-SDI setting the comorbidity pattern may reflect a cascade initiated by metabolic and dietary exposures, with reflux acting as one potential mediator rather than the sole driver.

However, it is important to note that in the SEM analysis, the contemporaneous path coefficient from GERD to asthma was negative. In contrast, the Granger causality test supported a statistically significant temporal sequence, suggesting that reflux precedes respiratory symptoms. The negative SEM path may therefore reflect complex time-varying and contemporaneous relationships captured by ecological time-series data rather than a direct protective biological effect. One plausible explanation is differential treatment and symptom suppression: acid-suppressive therapy in patients with severe reflux may reduce acute asthma exacerbations (38) or alter symptom reporting, which could manifest as an inverse association in cross-sectional snapshots. Tropical Latin America exhibits persistently high GERD prevalence (5) and may experience heterogeneous access to appropriate long-term management, together with widespread and often inappropriate PPI use (39). Thus, although GERD may act as a temporal trigger, optimising reflux management could still help disrupt the comorbidity cycle in susceptible populations.

The comorbidity trend in High-income North America presents a distinct “scissoring” or divergence pattern: asthma prevalence continues to rise, while the GERD burden has shown the most significant declining trend over the past three decades (AAPC = −0.33). The decline in GERD burden is likely attributable to advantages of a high-SDI society, including earlier diagnosis, effective symptom control, and access to medications, particularly PPIs (39). However, interaction NBR models suggested that several metabolic and dietary exposures (including fasting plasma glucose) were inversely associated with GERD in High-income North America, whereas their associations with asthma were weaker or directionally different compared with the other regions. This pattern is more consistent with healthcare-driven measurement and management effects (“medicalization”) than with true biological protection, because acid suppression may reduce symptomatic GERD without addressing the broader drivers of asthma burden emphasised by contemporary asthma strategies (40). Accordingly, the bidirectional Granger causality observed here may indicate that within a highly medicalised environment the residual comorbidity represents a more complex, treatment-modified cycle rather than a simple natural disease progression.

Interestingly, several well-established pathogenic factors, such as Occupational asthmagens, were not identified as positive predictors in our models-a finding that appears to contradict common clinical knowledge. This discrepancy likely reflects an ecological fallacy. In high-income regions (e.g., North America), despite carrying a substantial asthma burden, the shift to service-based economies and the implementation of stringent occupational safety regulations (41) have led to relatively low population-level exposure to these agents. Consequently, our population-level models may capture socioeconomic patterning of exposures rather than individual-level causal effects. This suggests that while occupational risks remain crucial for specific trades, their overall driving force at the macro level may be outweighed by metabolic and lifestyle factors.

Furthermore, we observed that some factors identified as positive influencers in the SHAP interpretability analysis showed inverse associations (RR < 1) in the pooled multivariable NBR models. This discrepancy can arise from methodological and structural differences: random forests capture nonlinearities and interactions, whereas NBR estimates average log-linear rate ratios after covariate adjustment. In addition, strong regional effect modification and development-related confounding may yield apparent “protective” associations in high-SDI settings where diagnosis, treatment, and symptom control vary over time. Our interaction analyses support this interpretation by demonstrating widespread heterogeneity of exposure-outcome associations across regions (20). Therefore, negative pooled coefficients should be interpreted cautiously as context-dependent population-level associations rather than evidence against biological plausibility.

## 5 Conclusion

In summary, the GERD-asthma comorbidity presents a multifaceted challenge fueled by shared dietary and metabolic factors. Its evolutionary trajectory is profoundly influenced by the regional Socio-demographic Index (SDI) context. Consequently, the development and implementation of rational and effective policies to mitigate this dual disease burden must be tailored to local conditions.

### 5.1 Limitations

This study also inevitably has several limitations. First, the associations observed at the population level cannot be directly extrapolated to individual-level biological causality. The regional heterogeneity we identified reflects characteristics of the macro-epidemiologic transition more than it defines individual clinical risk profiles. Second, although the GBD provides the most comprehensive global estimates, its accuracy is contingent upon the quality of original registration data from each region. Furthermore, as this study analysed only three representative regions, associated biases are unavoidable. Third, although Granger causality tests and SEM path analysis offer certain statistical evidence, they indicate predictive causality rather than definitive biological proof. Consequently, this research serves as a preliminary exploratory analysis of the current comorbidity patterns. Future work should involve more extensive global macro-epidemiological studies, as well as refined individual-level longitudinal cohort studies incorporating genetic and microbiome biomarkers, to further elucidate the GERD-asthma comorbidity patterns and regional phenotypes.

## Data Availability

The original contributions presented in the study are included in the article/Supplementary material, further inquiries can be directed to the corresponding author.

## Footnotes

1
https://vizhub.healthdata.org/gbd-results/

## References

[1] Dunbar KB. Gastroesophageal reflux disease. Ann Intern Med. (2024) 177:Itc113–itc28. doi: 10.7326/AITC202408200 39133924

[2] Miller RL Grayson MH Strothman K. Advances in asthma: new understandings of asthma's natural history, risk factors, underlying mechanisms, and clinical management. J Allergy Clin Immunol. (2021) 148:1430–41. doi: 10.1016/j.jaci.2021.10.001, 34655640

[3] Yuan L Tao J Wang J She W Zou Y Li R . Global, regional, national burden of asthma from 1990 to 2021, with projections of incidence to 2050: a systematic analysis of the global burden of disease study 2021. EClinicalMedicine. (2025) 80:103051. doi: 10.1016/j.eclinm.2024.103051, 39867965 PMC11764843

[4] Zhang D Liu S Li Z Wang R. Global, regional and national burden of gastroesophageal reflux disease, 1990-2019: update from the GBD 2019 study. Ann Med. (2022) 54:1372–84. doi: 10.1080/07853890.2022.2074535, 35579516 PMC9122392

[5] Li N Yang WL Cai MH Chen X Zhao R Li MT . Burden of gastroesophageal reflux disease in 204 countries and territories, 1990-2019: a systematic analysis for the global burden of disease study 2019. BMC Public Health. (2023) 23:582. doi: 10.1186/s12889-023-15272-z, 36978027 PMC10053627

[6] Mallah N Turner JM González-Barcala FJ Takkouche B. Gastroesophageal reflux disease and asthma exacerbation: a systematic review and meta-analysis. Pediatr Allergy Immunol. (2022) 33:e13655. doi: 10.1111/pai.13655, 34448255

[7] Zheng K Wang X Tang L Chen L Zhao Y Chen X. A systematic review and meta-analysis exploring the bidirectional association between asthma and gastroesophageal reflux disease in children. Allergy Asthma Proc. (2024) 45:e101–10. doi: 10.2500/aap.2024.45.240085, 39517072

[8] Broers C Tack J Pauwels A. Review article: gastro-oesophageal reflux disease in asthma and chronic obstructive pulmonary disease. Aliment Pharmacol Ther. (2018) 47:176–91. doi: 10.1111/apt.14416 29193245

[9] Bongiovanni A Parisi GF Scuderi MG Licari A Brambilla I Marseglia GL . Gastroesophageal reflux and respiratory diseases: does a real link exist? Minerva Pediatr. (2019) 71:515–23. doi: 10.23736/s0026-4946.19.05531-2, 31129955

[10] Iqbal N Amirali A Lail GU Khan MA Sial R Irfan M. Correlation of gastro-esophageal reflux disease with asthma control and quality of life: a cross-sectional study from a low-middle income country. Ther Adv Respir Dis. (2024) 18:17534666241297879. doi: 10.1177/17534666241297879, 39512235 PMC11544649

[11] Lee AS Lee JS He Z Ryu JH. Reflux-aspiration in chronic lung disease. Ann Am Thorac Soc. (2020) 17:155–64. doi: 10.1513/AnnalsATS.201906-427CME, 31697575

[12] Kopsaftis Z Yap HS Tin KS Hnin K Carson-Chahhoud KV. Pharmacological and surgical interventions for the treatment of gastro-oesophageal reflux in adults and children with asthma. Cochrane Database Syst Rev. (2021) 5:Cd001496. doi: 10.1002/14651858.CD001496.pub2, 33998673 PMC8127576

[13] Lee AS Ryu JH. Aspiration pneumonia and related syndromes. Mayo Clin Proc. (2018) 93:752–62. doi: 10.1016/j.mayocp.2018.03.011, 29730088

[14] Okwara NC Chan WW. Sorting out the relationship between esophageal and pulmonary disease. Gastroenterol Clin N Am. (2021) 50:919–34. doi: 10.1016/j.gtc.2021.08.006, 34717879

[15] Farah CS Salome CM. Asthma and obesity: a known association but unknown mechanism. Respirology. (2012) 17:412–21. doi: 10.1111/j.1440-1843.2011.02080.x, 21992497

[16] Festi D Scaioli E Baldi F Vestito A Pasqui F Biase ARD . Body weight, lifestyle, dietary habits and gastroesophageal reflux disease. World J Gastroenterol. (2009) 15:1690–701. doi: 10.3748/wjg.15.1690, 19360912 PMC2668774

[17] De Filippis A Ullah H Baldi A Dacrema M Esposito C Garzarella EU . Gastrointestinal disorders and metabolic syndrome: dysbiosis as a key link and common bioactive dietary components useful for their treatment. Int J Mol Sci. (2020) 21:32668581. doi: 10.3390/ijms21144929, 32668581 PMC7404341

[18] Lv N Xiao L Camargo CA Jr Wilson SR Buist AS Strub P . Abdominal and general adiposity and level of asthma control in adults with uncontrolled asthma. Ann Am Thorac Soc. (2014) 11:1218–24. doi: 10.1513/AnnalsATS.201405-214OC, 25343191 PMC4299000

[19] Chen CC Geng JH Wu PY Huang JC Hu HM Chen SC . High obesity indices are associated with gastroesophageal reflux disease, but low obesity indices are associated with peptic ulcer disease in a large Taiwanese population study. Obes Facts. (2024) 17:491–501. doi: 10.1159/000540281, 39008955 PMC11458163

[20] Sudharsanan N Aburto JM Riffe T van Raalte A. Commentary: large variation in the epidemiological transition across countries: is it still valuable as a mortality theory? Int J Epidemiol. (2022) 51:1057–61. doi: 10.1093/ije/dyac107, 35639549 PMC9365622

[21] Hay SI Ong KL Santomauro DF. Burden of 375 diseases and injuries, risk-attributable burden of 88 risk factors, and healthy life expectancy in 204 countries and territories, including 660 subnational locations, 1990-2023: a systematic analysis for the global burden of disease study 2023. Lancet. (2025) 406:1873–922. doi: 10.1016/S0140-6736(25)01637-X, 41092926 PMC12535840

[22] Austin ES Peng Z Ryan MB Bhoomadevi A Aalipour MA Aalruz H . Global age-sex-specific all-cause mortality and life expectancy estimates for 204 countries and territories and 660 subnational locations, 1950-2023: a demographic analysis for the global burden of disease study 2023. Lancet. (2025) 406:1731–810. doi: 10.1016/S0140-6736(25)01330-3 41092927 PMC12535839

[23] Mohsen N Hmwe HK Bhoomadevi A Aalipour MA Aalruz H Ababneh HS . Global burden of 292 causes of death in 204 countries and territories and 660 subnational locations, 1990-2023: a systematic analysis for the global burden of disease study 2023. Lancet. (2025) 406:1811–72. doi: 10.1016/S0140-6736(25)01917-8 41092928 PMC12535838

[24] de Benedictis FM Guidi R Bush A. Reflux-aspiration in chronic lung disease. Ann Am Thorac Soc. (2020) 17:1030. doi: 10.1513/AnnalsATS.202002-110LE, 32437248 PMC7393788

[25] Kim HJ Fay MP Feuer EJ Midthune DN. Permutation tests for joinpoint regression with applications to cancer rates. Stat Med. (2000) 19:335–51. doi: 10.1002/(SICI)1097-0258(20000215)19:3<335::AID-SIM336>3.0.CO;2-Z, 10649300

[26] Hyndman RJ Athanasopoulos G. Forecasting: Principles and Practice. 3rd ed. OTexts: Melbourne, Australia (2021). Available online at: https://otexts.com/fpp3/

[27] Breiman L. Random forests. Mach Learn. (2001) 45:5–32. doi: 10.1023/a:1010933404324

[28] Lundberg SM Lee S-I. "A unified approach to interpreting model predictions". In: Proceedings of the 31st International Conference on Neural Information Processing Systems. Long Beach, California: Curran Associates Inc. (2017). p. 4768–77.

[29] An X Liu Z Zhang L Zhao J Gu Q Han W . Co-occurrence patterns and related risk factors of ischaemic heart disease and ischaemic stroke across 203 countries and territories: a spatial correspondence and systematic analysis. Lancet Glob Health. (2025) 13:e808–19. doi: 10.1016/S2214-109X(25)00013-0, 40288393

[30] Hilbe JM, editor. "Negative binomial regression: modeling". In: Negative Binomial Regression, 2nd Edn. Cambridge: Cambridge University Press (2011). p. 221–83.

[31] McCravy M Ingram JL Que LG. Dysregulated metabolism in the pathophysiology of non-allergic obese asthma. J Asthma Allergy. (2021) 14:179–86. doi: 10.2147/JAA.S282284, 33692628 PMC7939487

[32] Cao X Lu T Tu Y Zhou R Li X Du L. The association between adult asthma in the United States and dietary total energy intake: a retrospective cross-sectional analysis from NHANES. BMC Nutr. (2024) 10:128. doi: 10.1186/s40795-024-00938-7, 39334497 PMC11437793

[33] Emrani AS Sasanfar B Jowshan MR Behniafard N Nafei Z Salehi-Abargouei A. Association between a western diet and asthma among children and adolescents. Sci Rep. (2024) 14:13240. doi: 10.1038/s41598-024-64008-5, 38853175 PMC11162998

[34] Eusebi LH Ratnakumaran R Yuan Y Solaymani-Dodaran M Bazzoli F Ford AC. Global prevalence of, and risk factors for, gastro-oesophageal reflux symptoms: a meta-analysis. Gut. (2018) 67:430–40. doi: 10.1136/gutjnl-2016-313589, 28232473

[35] Mo L Liu Z Cao W Gong H Wu J Lin M . Global, regional, and national burden of gastroesophageal reflux disease (1990-2021): age-period-cohort analysis and Bayesian projections. Front Public Health. (2025) 13:1576527. doi: 10.3389/fpubh.2025.1576527, 40703162 PMC12283765

[36] Sun Z Zheng Y. Metabolic diseases in the east Asian populations. Nat Rev Gastroenterol Hepatol. (2025) 22:500–16. doi: 10.1038/s41575-025-01058-8, 40200111

[37] Havemann BD Henderson CA El-Serag HB. The association between gastro-oesophageal reflux disease and asthma: a systematic review. Gut. (2007) 56:1654–64. doi: 10.1136/gut.2007.122465, 17682001 PMC2095717

[38] Akaba T Jo T Tagaya E Yasunaga H. Relationship between proton pump inhibitor prescription and asthma exacerbation among adult patients: a self-controlled case series study. Intern Emerg Med. (2024) 19:1905–12. doi: 10.1007/s11739-024-03687-4, 38904742

[39] Targownik LE Fisher DA Saini SD. AGA clinical practice update on de-prescribing of proton pump inhibitors: expert review. Gastroenterology. (2022) 162:1334–42. doi: 10.1053/j.gastro.2021.12.247, 35183361

[40] Levy ML Bacharier LB Bateman E Boulet LP Brightling C Buhl R . Key recommendations for primary care from the 2022 global initiative for asthma (GINA) update. NPJ Prim Care Respir Med. (2023) 33:7. doi: 10.1038/s41533-023-00330-1, 36754956 PMC9907191

[41] Vanroelen C. Employment quality: An overlooked determinant of workers' health and well-being? Ann Work Expo Health. (2019) 63:619–23. doi: 10.1093/annweh/wxz049, 31225592

