# Supplementary material for: Ecological co-burden patterns of GERD and asthma: a multi-method analysis of representative global regions
Source: Front Nutr. 2026 Mar 26;13:1774196. doi: 10.3389/fnut.2026.1774196 (PMC13062313; doi:10.3389/fnut.2026.1774196)
Supplement: Supplementary file 6 [file Data_Sheet_6.pdf]

Supplementary Tables

Supplementary Table 1. Global and regional (21 regions) age-standardized prevalence rates and all-age prevalence numbers of Gastroesophageal Reflux Disease in 1994 and 2023

Region	1994 Rate per 100,000 (95% UI)	1994 Cases, million (95% UI)	2023 Rate per 100,000 (95% UI)	2023 Cases, million (95% UI)
Global	9,464.9 (8,458.5–10,633.9)	488.0 (433.9–554.1)	9,725.8 (8,680.0–10,947.0)	841.1 (751.6–943.0)
High-income North America	10,517.9 (9,249.3–11,886.0)	35.4 (30.9–39.8)	9,530.3 (8,359.3–10,778.3)	45.4 (40.2–51.1)
Australasia	8,774.8 (7,795.2–9,943.3)	2.1 (1.9–2.4)	8,697.3 (7,619.4–9,724.9)	3.5 (3.1–4.0)
High-income Asia Pacific	6,126.6 (5,414.0–6,992.0)	12.8 (11.3–14.5)	6,257.5 (5,557.1–7,060.3)	16.9 (14.9–19.2)
Western Europe	8,211.6 (7,268.2–9,273.7)	39.7 (35.4–44.5)	8,155.4 (7,228.1–9,219.8)	49.1 (43.7–55.4)
Southern Latin America	14,225.7 (12,793.0–15,819.2)	7.2 (6.5–8.1)	13,527.7 (11,956.2–15,237.9)	11.0 (9.7–12.4)
Andean Latin America	16,410.6 (14,510.1–18,366.7)	5.4 (4.8–6.1)	16,248.4 (14,363.3–18,185.2)	11.1 (9.8–12.4)
Central Latin America	16,423.9 (14,574.3–18,435.9)	23.4 (20.8–26.4)	16,286.8 (14,449.3–18,283.7)	45.2 (40.1–50.8)
Tropical Latin America	17,123.4 (15,290.5–19,190.8)	24.6 (22.0–28.0)	16,591.0 (14,822.0–18,610.6)	42.0 (37.4–46.8)
Caribbean	16,412.5 (14,512.5–18,367.7)	5.6 (5.0–6.3)	16,288.4 (14,400.3–18,227.1)	8.5 (7.6–9.5)
North Africa and Middle East	12,328.4 (10,921.3–13,859.8)	35.2 (31.2–40.1)	12,086.0 (10,692.7–13,627.5)	75.5 (66.7–85.4)
Central Europe	11,120.7 (9,855.2–12,447.7)	16.0 (14.2–17.9)	11,111.6 (9,834.1–12,434.1)	17.5 (15.7–19.5)

Eastern Europe	11,612.9 (10,292.5–13,044.9)	30.7 (27.1–34.6)	11,503.6 (10,189.4–12,929.2)	32.4 (28.6–36.4)
Central Asia	10,876.7 (9,523.2–12,256.9)	6.6 (5.7–7.5)	10,772.8 (9,432.1–12,138.5)	10.7 (9.3–12.2)
South Asia	13,661.4 (12,280.0–15,333.1)	124.2 (111.1–141.4)	13,423.2 (12,062.1–15,069.6)	248.4 (222.3–281.0)
East Asia	4,359.6 (3,842.0–4,960.5)	54.7 (47.8–62.6)	4,465.0 (3,928.9–5,048.7)	84.6 (74.0–95.8)
Southeast Asia	5,495.2 (4,848.0–6,224.5)	22.7 (19.7–26.0)	5,420.3 (4,786.5–6,142.2)	41.6 (36.5–47.1)
Oceania	5,326.2 (4,701.6–6,031.1)	0.3 (0.2–0.3)	5,292.3 (4,671.8–5,993.8)	0.6 (0.6–0.7)
Western Sub-Saharan Africa	11,621.6 (10,321.8–13,067.6)	16.6 (14.5–18.9)	11,442.1 (10,144.2–12,853.3)	40.0 (35.1–45.7)
Central Sub-Saharan Africa	11,407.5 (10,077.5–12,866.3)	4.6 (4.0–5.2)	11,209.3 (9,901.6–12,640.4)	11.2 (9.8–12.7)
Eastern Sub-Saharan Africa	11,588.1 (10,281.9–13,031.1)	15.2 (13.3–17.3)	11,391.9 (10,100.8–12,810.5)	35.7 (31.3–40.8)
Southern Sub-Saharan Africa	11,795.5 (10,486.7–13,191.0)	5.2 (4.5–5.9)	11,717.8 (10,422.7–13,099.6)	10.0 (8.8–11.3)

Rate: age-standardized prevalence rate per 100,000 (95% uncertainty interval). **Cases**: prevalent cases (all ages), in millions (95% uncertainty interval).

Supplementary Table 2. Global and regional (21 regions) age-standardized prevalence rates and all-age prevalence numbers of Asthma in 1994 and 2023

Region	1994 Rate per 100,000 (95% UI)	1994 Cases, million (95% UI)	2023 Rate per 100,000 (95% UI)	2023 Cases, million (95% UI)
Global	4,331.8 (3,687.1–5,104.4)	240.9 (203.5–286.0)	4,457.7 (3,786.9–5,246.9)	362.7 (309.8–427.6)
High-income North America	9,356.9 (7,840.6–11,107.0)	26.7 (22.4–31.7)	11,044.5 (9,327.4–13,130.2)	40.7 (35.0–47.8)
Australasia	13,219.8 (12,503.8–14,150.2)	2.7 (2.6–2.9)	13,364.4 (10,910.1–16,540.6)	4.1 (3.4–4.9)
High-income Asia Pacific	5,119.6 (4,130.2–6,386.8)	8.4 (6.9–10.2)	4,870.2 (4,046.7–5,962.8)	8.9 (7.3–10.4)
Western Europe	6,881.7 (6,042.4–8,074.8)	26.8 (23.4–31.0)	7,687.7 (6,600.2–9,187.8)	34.5 (29.6–40.3)
Southern Latin America	6,162.5 (5,130.1–7,387.0)	3.2 (2.7–3.9)	6,511.6 (5,417.7–7,768.4)	4.4 (3.7–5.2)
Andean Latin America	6,669.4 (5,436.8–8,069.9)	3.2 (2.6–3.9)	6,231.6 (5,078.7–7,594.7)	3.9 (3.2–4.8)
Central Latin America	6,221.4 (5,164.5–7,477.6)	11.9 (9.7–14.6)	6,418.0 (5,414.8–7,689.8)	16.3 (13.8–19.4)
Tropical Latin America	6,850.1 (5,619.4–8,357.1)	11.7 (9.4–14.4)	8,810.9 (7,264.5–10,813.7)	18.3 (15.2–22.1)
Caribbean	8,720.8 (7,236.4–10,588.1)	3.3 (2.7–4.1)	8,759.6 (7,252.7–10,529.7)	4.1 (3.5–4.9)
North Africa and Middle East	5,000.7 (4,168.8–6,151.1)	18.5 (15.3–23.1)	5,144.5 (4,353.8–6,213.7)	32.5 (27.5–39.3)
Central Europe	4,634.0 (3,871.0–5,564.9)	6.2 (5.1–7.4)	4,270.1 (3,535.8–5,190.3)	5.6 (4.5–6.7)
Eastern Europe	3,484.4 (2,865.9–4,275.2)	8.2 (6.7–10.0)	4,162.8 (3,423.8–5,125.9)	9.6 (7.7–11.6)

Central Asia	2,229.2 (1,834.7–2,707.0)	1.5 (1.3–1.9)	2,258.8 (1,830.2–2,775.4)	2.2 (1.8–2.8)
South Asia	2,884.7 (2,382.0–3,341.8)	26.7 (22.5–32.1)	2,475.2 (2,062.2–2,953.3)	44.4 (37.1–53.7)
East Asia	2,957.6 (2,438.4–3,593.4)	35.8 (29.3–43.7)	3,118.7 (2,577.3–3,732.2)	48.2 (39.0–56.8)
Southeast Asia	4,740.4 (3,954.7–5,687.9)	23.0 (19.0–28.2)	4,778.2 (3,970.3–5,735.6)	33.2 (27.7–39.7)
Oceania	6,825.2 (5,530.1–8,338.2)	0.5 (0.4–0.7)	6,834.1 (5,509.2–8,386.3)	1.0 (0.8–1.3)
Western Sub-Saharan Africa	3,348.3 (2,760.3–4,054.9)	7.5 (6.1–9.3)	3,302.9 (2,741.4–4,058.8)	17.6 (14.4–22.1)
Central Sub-Saharan Africa	5,306.9 (4,367.0–6,490.8)	3.4 (2.7–4.3)	5,553.4 (4,654.3–6,715.8)	8.6 (7.0–10.6)
Eastern Sub-Saharan Africa	4,292.4 (3,523.5–5,194.5)	9.6 (7.6–12.0)	4,323.5 (3,555.4–5,260.8)	20.7 (16.5–26.1)
Southern Sub-Saharan Africa	3,726.5 (3,134.4–4,529.1)	2.2 (1.8–2.7)	4,182.3 (3,475.7–5,149.1)	3.7 (3.0–4.6)

Rate: age-standardized prevalence rate per 100,000 (95% uncertainty interval). **Cases**: prevalent cases (all ages), in millions (95% uncertainty interval).

Inclusion Criteria for Regional Selection in the Present Study: Firstly, East Asia exhibits the globally lowest age-standardized rate of GERD, yet carries an extremely high absolute case number (4,465.0, 95%UI:3,928.9–5,048.7). This indicates a vast affected population, with the phenomenon potentially attributable to a significant age-related disease burden. Secondly, Tropical Latin America has the world's highest age-standardized prevalence rate(16,591.0, 95%UI:14,822.0–18,610.6). Finally, High-income North America shows the most pronounced declining trend in GERD burden over the past three decades[1,2]. These three regions also correspond to different Socio-demographic Index (SDI) levels, which allows for an indirect assessment of the impact of societal development on disease burden

Supplement Table 3: Average Annual Percent Change (AAPC) of the Prevalence of Gastroesophageal Reflux Disease (GERD) and Asthma in Three Regions

Measure	Location	Sex	Cause	Joinpoint	Start.Obs	End.Obs	P.Value	Metric	Age	AAPC(95%CI)
Prevalence	East Asia	Both	Asthma	5	1994	2023	0	Rate	Age-standardized	0.22 (0.16 to 0.28)
Prevalence	East Asia	Both	GERD	4	1994	2023	0	Rate	Age-standardized	0.1 (0.07 to 0.13)
Prevalence	High-income North America	Both	Asthma	5	1994	2023	0	Rate	Age-standardized	0.57 (0.53 to 0.61)
Prevalence	High-income North America	Both	GERD	4	1994	2023	0	Rate	Age-standardized	-0.33 (-0.36 to -0.31)
Prevalence	Tropical Latin America	Both	Asthma	3	1994	2023	0	Rate	Age-standardized	1.05 (0.89 to 1.21)
Prevalence	Tropical Latin America	Both	GERD	2	1994	2023	0	Rate	Age-standardized	-0.09 (-0.1 to -0.08)

Supplement Table 4: Annual Percent Change (APC) of the Prevalence of Gastroesophageal Reflux Disease (GERD) and Asthma in Three Regions

Measure	Location	Sex	Cause	Joinpoint	Segment	Segment.Start	Segment.End	P.Value	Metric	Age	APC(95%CI)
Prevalence	East Asia	Both	Asthma	5	0	1994	1996	0.00	Rate	Age-standardized	-2.52 (-3.77 to -1.55)
Prevalence	East Asia	Both	Asthma	5	1	1996	2000	0.00	Rate	Age-standardized	-6.12 (-6.62 to -5.76)
Prevalence	East Asia	Both	Asthma	5	2	2000	2005	0.00	Rate	Age-standardized	2.67 (2.3 to 3.09)
Prevalence	East Asia	Both	Asthma	5	3	2005	2010	0.02	Rate	Age-standardized	-0.56 (-1.27 to -0.1)
Prevalence	East Asia	Both	Asthma	5	4	2010	2015	0.03	Rate	Age-standardized	0.55 (0.1 to 2.18)
Prevalence	East Asia	Both	Asthma	5	5	2015	2023	0.00	Rate	Age-standardized	3 (2.77 to 3.32)
Prevalence	East Asia	Both	GERD	4	0	1994	2005	0.18	Rate	Age-standardized	0.04 (-0.03 to 0.11)
Prevalence	East Asia	Both	GERD	4	1	2005	2010	0.00	Rate	Age-standardized	-1.51 (-1.8 to -1.25)
Prevalence	East Asia	Both	GERD	4	2	2010	2015	0.02	Rate	Age-standardized	0.54 (0.19 to 0.83)
Prevalence	East Asia	Both	GERD	4	3	2015	2020	0.00	Rate	Age-standardized	1.96 (1.74 to 2.33)
Prevalence	East Asia	Both	GERD	4	4	2020	2023	0.01	Rate	Age-standardized	-0.75 (-1.13 to -0.33)
Prevalence	High-income North America	Both	Asthma	5	0	1994	1996	0.00	Rate	Age-standardized	0.92 (0.41 to 1.5)
Prevalence	High-income North America	Both	Asthma	5	1	1996	1999	0.00	Rate	Age-standardized	3.25 (2.96 to 3.54)
Prevalence	High-income North America	Both	Asthma	5	2	1999	2010	0.01	Rate	Age-standardized	0.1 (0.03 to 0.17)
Prevalence	High-income North America	Both	Asthma	5	3	2010	2015	0.00	Rate	Age-standardized	-1.28 (-1.6 to -1.04)
Prevalence	High-income North America	Both	Asthma	5	4	2015	2021	0.00	Rate	Age-standardized	0.52 (0.35 to 0.73)
Prevalence	High-income North America	Both	Asthma	5	5	2021	2023	0.00	Rate	Age-standardized	3.72 (3.1 to 4.2)
Prevalence	High-income North America	Both	GERD	4	0	1994	2001	0.14	Rate	Age-standardized	0.07 (-0.02 to 0.17)
Prevalence	High-income North America	Both	GERD	4	1	2001	2009	0.00	Rate	Age-standardized	-2.58 (-2.67 to -2.51)
Prevalence	High-income North America	Both	GERD	4	2	2009	2012	0.04	Rate	Age-standardized	0.38 (0.12 to 0.68)
Prevalence	High-income North America	Both	GERD	4	3	2012	2019	0.00	Rate	Age-standardized	1.42 (1.31 to 1.64)
Prevalence	High-income North America	Both	GERD	4	4	2019	2023	0.64	Rate	Age-standardized	-0.04 (-0.33 to 0.25)
Prevalence	Tropical Latin America	Both	Asthma	3	0	1994	2000	0.37	Rate	Age-standardized	0.26 (-0.48 to 2.13)
Prevalence	Tropical Latin America	Both	Asthma	3	1	2000	2005	0.01	Rate	Age-standardized	-2.8 (-4.85 to -1.75)
Prevalence	Tropical Latin America	Both	Asthma	3	2	2005	2020	0.00	Rate	Age-standardized	0.87 (0.63 to 1.13)
Prevalence	Tropical Latin America	Both	Asthma	3	3	2020	2023	0.00	Rate	Age-standardized	10.46 (7.76 to 15.16)
Prevalence	Tropical Latin America	Both	GERD	2	0	1994	2006	0.01	Rate	Age-standardized	-0.05 (-0.07 to -0.02)
Prevalence	Tropical Latin America	Both	GERD	2	1	2006	2009	0.00	Rate	Age-standardized	-0.67 (-0.76 to -0.45)
Prevalence	Tropical Latin America	Both	GERD	2	2	2009	2023	0.34	Rate	Age-standardized	-0.01 (-0.03 to 0.02)

Supplement Table 5: Predicted prevalence of gastroesophageal reflux disease (GERD) and asthma in three regions: 2024-2033

Region	Disease	Year	Rate	Type	Lower	Upper
East Asia	GERD	1994	4359.636	Historical	NA	NA
East Asia	GERD	1995	4342.675	Historical	NA	NA
East Asia	GERD	1996	4342.394	Historical	NA	NA
East Asia	GERD	1997	4352.363	Historical	NA	NA
East Asia	GERD	1998	4366.274	Historical	NA	NA
East Asia	GERD	1999	4378.129	Historical	NA	NA
East Asia	GERD	2000	4382.075	Historical	NA	NA
East Asia	GERD	2001	4379.641	Historical	NA	NA
East Asia	GERD	2002	4376.029	Historical	NA	NA
East Asia	GERD	2003	4370.753	Historical	NA	NA
East Asia	GERD	2004	4363.464	Historical	NA	NA
East Asia	GERD	2005	4353.789	Historical	NA	NA
East Asia	GERD	2006	4317.549	Historical	NA	NA
East Asia	GERD	2007	4247.117	Historical	NA	NA
East Asia	GERD	2008	4166.592	Historical	NA	NA
East Asia	GERD	2009	4100.248	Historical	NA	NA
East Asia	GERD	2010	4072.138	Historical	NA	NA
East Asia	GERD	2011	4077.298	Historical	NA	NA
East Asia	GERD	2012	4092.798	Historical	NA	NA
East Asia	GERD	2013	4115.553	Historical	NA	NA
East Asia	GERD	2014	4142.637	Historical	NA	NA
East Asia	GERD	2015	4171.120	Historical	NA	NA
East Asia	GERD	2016	4229.203	Historical	NA	NA
East Asia	GERD	2017	4326.875	Historical	NA	NA
East Asia	GERD	2018	4433.732	Historical	NA	NA
East Asia	GERD	2019	4519.555	Historical	NA	NA
East Asia	GERD	2020	4554.300	Historical	NA	NA
East Asia	GERD	2021	4553.489	Historical	NA	NA
East Asia	GERD	2022	4552.677	Historical	NA	NA
East Asia	GERD	2023	4465.014	Historical	NA	NA
East Asia	GERD	2024	4377.368	Forecast	4322.670	4432.066
East Asia	GERD	2025	4289.713	Forecast	4167.899	4411.528
East Asia	GERD	2026	4202.059	Forecast	3999.148	4404.969
East Asia	GERD	2027	4114.404	Forecast	3818.774	4410.035
East Asia	GERD	2028	4026.750	Forecast	3628.382	4425.118
East Asia	GERD	2029	3939.095	Forecast	3429.152	4449.038
East Asia	GERD	2030	3851.440	Forecast	3222.000	4480.881
East Asia	GERD	2031	3763.786	Forecast	3007.660	4519.911
East Asia	GERD	2032	3676.131	Forecast	2786.745	4565.517
East Asia	GERD	2033	3588.477	Forecast	2559.770	4617.183
East Asia	Asthma	1994	2957.644	Historical	NA	NA
East Asia	Asthma	1995	2926.684	Historical	NA	NA
East Asia	Asthma	1996	2832.644	Historical	NA	NA
East Asia	Asthma	1997	2658.299	Historical	NA	NA
East Asia	Asthma	1998	2461.344	Historical	NA	NA
East Asia	Asthma	1999	2299.486	Historical	NA	NA
East Asia	Asthma	2000	2230.153	Historical	NA	NA
East Asia	Asthma	2001	2251.622	Historical	NA	NA
East Asia	Asthma	2002	2309.463	Historical	NA	NA
East Asia	Asthma	2003	2381.954	Historical	NA	NA
East Asia	Asthma	2004	2447.758	Historical	NA	NA
East Asia	Asthma	2005	2485.575	Historical	NA	NA
East Asia	Asthma	2006	2491.030	Historical	NA	NA
East Asia	Asthma	2007	2480.323	Historical	NA	NA
East Asia	Asthma	2008	2461.841	Historical	NA	NA
East Asia	Asthma	2009	2444.328	Historical	NA	NA
East Asia	Asthma	2010	2436.344	Historical	NA	NA
East Asia	Asthma	2011	2441.276	Historical	NA	NA
East Asia	Asthma	2012	2455.063	Historical	NA	NA
East Asia	Asthma	2013	2474.152	Historical	NA	NA
East Asia	Asthma	2014	2495.151	Historical	NA	NA
East Asia	Asthma	2015	2514.695	Historical	NA	NA
East Asia	Asthma	2016	2556.575	Historical	NA	NA
East Asia	Asthma	2017	2634.505	Historical	NA	NA
East Asia	Asthma	2018	2729.800	Historical	NA	NA
East Asia	Asthma	2019	2823.820	Historical	NA	NA
East Asia	Asthma	2020	2897.560	Historical	NA	NA
East Asia	Asthma	2021	3015.020	Historical	NA	NA
East Asia	Asthma	2022	3109.372	Historical	NA	NA
East Asia	Asthma	2023	3118.710	Historical	NA	NA
East Asia	Asthma	2024	3128.063	Forecast	3046.156	3209.970
East Asia	Asthma	2025	3137.408	Forecast	2954.273	3320.543
East Asia	Asthma	2026	3146.752	Forecast	2840.312	3453.192
East Asia	Asthma	2027	3156.097	Forecast	2707.516	3604.677
East Asia	Asthma	2028	3165.441	Forecast	2558.062	3772.821
East Asia	Asthma	2029	3174.786	Forecast	2393.519	3956.053
East Asia	Asthma	2030	3184.130	Forecast	2215.089	4153.172
East Asia	Asthma	2031	3193.475	Forecast	2023.724	4363.226
East Asia	Asthma	2032	3202.820	Forecast	1820.206	4585.433
East Asia	Asthma	2033	3212.164	Forecast	1605.191	4819.138

East Asia	Asthma	2031	3193.475	Forecast	2023.724	4363.226
East Asia	Asthma	2032	3202.820	Forecast	1820.206	4585.433
East Asia	Asthma	2033	3212.164	Forecast	1605.191	4819.138
Tropical Latin America	GERD	1994	17123.403	Historical	NA	NA
Tropical Latin America	GERD	1995	17147.583	Historical	NA	NA
Tropical Latin America	GERD	1996	17144.322	Historical	NA	NA
Tropical Latin America	GERD	1997	17126.815	Historical	NA	NA
Tropical Latin America	GERD	1998	17103.881	Historical	NA	NA
Tropical Latin America	GERD	1999	17083.388	Historical	NA	NA
Tropical Latin America	GERD	2000	17073.921	Historical	NA	NA
Tropical Latin America	GERD	2001	17072.655	Historical	NA	NA
Tropical Latin America	GERD	2002	17071.471	Historical	NA	NA
Tropical Latin America	GERD	2003	17070.225	Historical	NA	NA
Tropical Latin America	GERD	2004	17069.152	Historical	NA	NA
Tropical Latin America	GERD	2005	17068.125	Historical	NA	NA
Tropical Latin America	GERD	2006	17027.987	Historical	NA	NA
Tropical Latin America	GERD	2007	16934.049	Historical	NA	NA
Tropical Latin America	GERD	2008	16822.047	Historical	NA	NA
Tropical Latin America	GERD	2009	16728.137	Historical	NA	NA
Tropical Latin America	GERD	2010	16688.242	Historical	NA	NA
Tropical Latin America	GERD	2011	16687.408	Historical	NA	NA
Tropical Latin America	GERD	2012	16686.639	Historical	NA	NA
Tropical Latin America	GERD	2013	16685.941	Historical	NA	NA
Tropical Latin America	GERD	2014	16685.329	Historical	NA	NA
Tropical Latin America	GERD	2015	16684.642	Historical	NA	NA
Tropical Latin America	GERD	2016	16683.819	Historical	NA	NA
Tropical Latin America	GERD	2017	16682.872	Historical	NA	NA
Tropical Latin America	GERD	2018	16682.536	Historical	NA	NA
Tropical Latin America	GERD	2019	16683.312	Historical	NA	NA
Tropical Latin America	GERD	2020	16685.882	Historical	NA	NA
Tropical Latin America	GERD	2021	16723.847	Historical	NA	NA
Tropical Latin America	GERD	2022	16746.041	Historical	NA	NA
Tropical Latin America	GERD	2023	16591.000	Historical	NA	NA
Tropical Latin America	GERD	2024	16591.015	Forecast	16499.291	16682.739
Tropical Latin America	GERD	2025	16591.015	Forecast	16461.305	16720.726
Tropical Latin America	GERD	2026	16591.015	Forecast	16432.156	16749.875
Tropical Latin America	GERD	2027	16591.015	Forecast	16407.582	16774.449
Tropical Latin America	GERD	2028	16591.015	Forecast	16385.931	16796.099
Tropical Latin America	GERD	2029	16591.015	Forecast	16366.358	16815.673
Tropical Latin America	GERD	2030	16591.015	Forecast	16348.358	16833.673
Tropical Latin America	GERD	2031	16591.015	Forecast	16331.604	16850.426
Tropical Latin America	GERD	2032	16591.015	Forecast	16315.868	16866.162
Tropical Latin America	GERD	2033	16591.015	Forecast	16300.985	16881.045
Tropical Latin America	Asthma	1994	6850.128	Historical	NA	NA
Tropical Latin America	Asthma	1995	6857.791	Historical	NA	NA
Tropical Latin America	Asthma	1996	6868.247	Historical	NA	NA
Tropical Latin America	Asthma	1997	6898.620	Historical	NA	NA
Tropical Latin America	Asthma	1998	6929.337	Historical	NA	NA
Tropical Latin America	Asthma	1999	6940.519	Historical	NA	NA
Tropical Latin America	Asthma	2000	6912.618	Historical	NA	NA
Tropical Latin America	Asthma	2001	6794.518	Historical	NA	NA
Tropical Latin America	Asthma	2002	6591.106	Historical	NA	NA
Tropical Latin America	Asthma	2003	6366.675	Historical	NA	NA
Tropical Latin America	Asthma	2004	6185.164	Historical	NA	NA
Tropical Latin America	Asthma	2005	6110.406	Historical	NA	NA
Tropical Latin America	Asthma	2006	6121.818	Historical	NA	NA
Tropical Latin America	Asthma	2007	6149.672	Historical	NA	NA
Tropical Latin America	Asthma	2008	6183.795	Historical	NA	NA
Tropical Latin America	Asthma	2009	6214.343	Historical	NA	NA
Tropical Latin America	Asthma	2010	6231.319	Historical	NA	NA
Tropical Latin America	Asthma	2011	6270.502	Historical	NA	NA
Tropical Latin America	Asthma	2012	6356.602	Historical	NA	NA
Tropical Latin America	Asthma	2013	6463.479	Historical	NA	NA
Tropical Latin America	Asthma	2014	6564.721	Historical	NA	NA
Tropical Latin America	Asthma	2015	6634.077	Historical	NA	NA
Tropical Latin America	Asthma	2016	6671.810	Historical	NA	NA
Tropical Latin America	Asthma	2017	6696.926	Historical	NA	NA
Tropical Latin America	Asthma	2018	6711.310	Historical	NA	NA
Tropical Latin America	Asthma	2019	6716.527	Historical	NA	NA
Tropical Latin America	Asthma	2020	6713.999	Historical	NA	NA
Tropical Latin America	Asthma	2021	7814.992	Historical	NA	NA
Tropical Latin America	Asthma	2022	8892.969	Historical	NA	NA
Tropical Latin America	Asthma	2023	8810.923	Historical	NA	NA
Tropical Latin America	Asthma	2024	8810.931	Forecast	8078.374	9543.489
Tropical Latin America	Asthma	2025	8810.931	Forecast	7774.525	9847.338
Tropical Latin America	Asthma	2026	8810.931	Forecast	7541.048	10080.815
Tropical Latin America	Asthma	2027	8810.931	Forecast	7343.949	10277.914
Tropical Latin America	Asthma	2028	8810.931	Forecast	7170.065	10451.797
Tropical Latin America	Asthma	2029	8810.931	Forecast	7012.649	10609.213
Tropical Latin America	Asthma	2030	8810.931	Forecast	6867.694	10754.168

High-income North America	GERD	1994	10517.934	Historical	NA	NA
High-income North America	GERD	1995	10506.834	Historical	NA	NA
High-income North America	GERD	1996	10517.769	Historical	NA	NA
High-income North America	GERD	1997	10549.719	Historical	NA	NA
High-income North America	GERD	1998	10587.916	Historical	NA	NA
High-income North America	GERD	1999	10617.738	Historical	NA	NA
High-income North America	GERD	2000	10624.437	Historical	NA	NA
High-income North America	GERD	2001	10529.836	Historical	NA	NA
High-income North America	GERD	2002	10307.166	Historical	NA	NA
High-income North America	GERD	2003	10018.452	Historical	NA	NA
High-income North America	GERD	2004	9725.942	Historical	NA	NA
High-income North America	GERD	2005	9491.747	Historical	NA	NA
High-income North America	GERD	2006	9278.303	Historical	NA	NA
High-income North America	GERD	2007	9036.041	Historical	NA	NA
High-income North America	GERD	2008	8808.813	Historical	NA	NA
High-income North America	GERD	2009	8640.155	Historical	NA	NA
High-income North America	GERD	2010	8573.865	Historical	NA	NA
High-income North America	GERD	2011	8607.366	Historical	NA	NA
High-income North America	GERD	2012	8693.838	Historical	NA	NA
High-income North America	GERD	2013	8810.861	Historical	NA	NA
High-income North America	GERD	2014	8935.968	Historical	NA	NA
High-income North America	GERD	2015	9046.636	Historical	NA	NA
High-income North America	GERD	2016	9164.717	Historical	NA	NA
High-income North America	GERD	2017	9307.443	Historical	NA	NA
High-income North America	GERD	2018	9445.350	Historical	NA	NA
High-income North America	GERD	2019	9549.119	Historical	NA	NA
High-income North America	GERD	2020	9589.295	Historical	NA	NA
High-income North America	GERD	2021	9587.822	Historical	NA	NA
High-income North America	GERD	2022	9586.734	Historical	NA	NA
High-income North America	GERD	2023	9530.335	Historical	NA	NA
High-income North America	GERD	2024	9474.281	Forecast	9352.396	9596.166
High-income North America	GERD	2025	9418.055	Forecast	9146.511	9689.599
High-income North America	GERD	2026	9361.829	Forecast	8908.234	9815.424
High-income North America	GERD	2027	9305.603	Forecast	8642.597	9968.609
High-income North America	GERD	2028	9249.377	Forecast	8352.947	10145.807
High-income North America	GERD	2029	9193.151	Forecast	8041.700	10344.602
High-income North America	GERD	2030	9136.925	Forecast	7710.704	10563.146
High-income North America	GERD	2031	9080.699	Forecast	7361.427	10799.971
High-income North America	GERD	2032	9024.473	Forecast	6995.073	11053.873
High-income North America	GERD	2033	8968.247	Forecast	6612.647	11323.847
High-income North America	Asthma	1994	9356.897	Historical	NA	NA
High-income North America	Asthma	1995	9382.937	Historical	NA	NA
High-income North America	Asthma	1996	9532.852	Historical	NA	NA
High-income North America	Asthma	1997	9814.989	Historical	NA	NA
High-income North America	Asthma	1998	10136.540	Historical	NA	NA
High-income North America	Asthma	1999	10404.926	Historical	NA	NA
High-income North America	Asthma	2000	10527.266	Historical	NA	NA
High-income North America	Asthma	2001	10534.341	Historical	NA	NA
High-income North America	Asthma	2002	10525.854	Historical	NA	NA
High-income North America	Asthma	2003	10510.919	Historical	NA	NA
High-income North America	Asthma	2004	10498.112	Historical	NA	NA
High-income North America	Asthma	2005	10497.037	Historical	NA	NA
High-income North America	Asthma	2006	10510.418	Historical	NA	NA
High-income North America	Asthma	2007	10528.965	Historical	NA	NA
High-income North America	Asthma	2008	10548.674	Historical	NA	NA
High-income North America	Asthma	2009	10564.546	Historical	NA	NA
High-income North America	Asthma	2010	10570.665	Historical	NA	NA
High-income North America	Asthma	2011	10505.230	Historical	NA	NA
High-income North America	Asthma	2012	10349.743	Historical	NA	NA
High-income North America	Asthma	2013	10165.394	Historical	NA	NA
High-income North America	Asthma	2014	10012.928	Historical	NA	NA
High-income North America	Asthma	2015	9952.881	Historical	NA	NA
High-income North America	Asthma	2016	9970.005	Historical	NA	NA
High-income North America	Asthma	2017	10005.300	Historical	NA	NA
High-income North America	Asthma	2018	10053.458	Historical	NA	NA
High-income North America	Asthma	2019	10109.163	Historical	NA	NA
High-income North America	Asthma	2020	10167.150	Historical	NA	NA
High-income North America	Asthma	2021	10300.144	Historical	NA	NA
High-income North America	Asthma	2022	10537.682	Historical	NA	NA
High-income North America	Asthma	2023	11044.480	Historical	NA	NA
High-income North America	Asthma	2024	11551.235	Forecast	11357.018	11745.451
High-income North America	Asthma	2025	12058.016	Forecast	11619.880	12496.152
High-income North America	Asthma	2026	12564.797	Forecast	11824.190	13305.405
High-income North America	Asthma	2027	13071.579	Forecast	11975.968	14167.190
High-income North America	Asthma	2028	13578.360	Forecast	12078.958	15077.762
High-income North America	Asthma	2029	14085.142	Forecast	12135.617	16034.667
High-income North America	Asthma	2030	14591.923	Forecast	12147.613	17036.233
High-income North America	Asthma	2031	15098.704	Forecast	12116.103	18081.305
High-income North America	Asthma	2032	15605.486	Forecast	12041.882	19169.089
High-income North America	Asthma	2033	16112.267	Forecast	11925.484	20299.050

Supplementary Table 6: Negative Binomial Regression of Risk Factors for Asthma and Gastroesophageal Reflux Disease (GERD)

Negative Binomial Regression Analysis of Asthma Risk Factors				
**Risk Factors**	**RR**	**Lower**	**Upper**	***P***
Ambient Ozone Pollution	1.010	0.958	1.065	0.723
Suboptimal Breastfeeding	1.364	1.227	1.516	0.000
High Fasting Plasma Glucose	1.133	1.075	1.195	0.000
High Body Mass Index	1.235	0.957	1.594	0.105
Diet Low In Vegetables	1.561	1.106	2.203	0.011
Diet High In Red Meat	1.662	1.505	1.836	0.000
Diet High In Processed Meat	0.579	0.321	1.045	0.070
Diet High In Sugar Sweetened Beverages	1.248	0.909	1.714	0.170
Low Physical Activity	1.163	1.018	1.329	0.027
High Temperature	1.011	0.989	1.034	0.332
Low Temperature	1.014	0.949	1.084	0.677
**Negative Binomial Regression Analysis of Gastroesophageal Reflux Disease(GERD) Risk Factors**				
**Risk Factors**	**RR**	**Lower**	**Upper**	***P***
Ambient Ozone Pollution	0.995	0.976	1.014	0.590
Suboptimal Breastfeeding	1.342	1.266	1.423	0.000
High Fasting Plasma Glucose	1.028	1.001	1.056	0.046
High Body Mass Index	0.671	0.584	0.771	0.000
Diet Low In Vegetables	0.827	0.723	0.946	0.006
Diet High In Red Meat	0.863	0.821	0.909	0.000
Diet High In Processed Meat	0.409	0.276	0.605	0.000
Diet High In Sugar Sweetened Beverages	1.505	1.247	1.817	0.000
Low Physical Activity	0.898	0.847	0.951	0.000
High Temperature	1.001	0.991	1.012	0.817
Low Temperature	0.972	0.943	1.002	0.069

Supplementary Table 7: Negative Binomial Regression of Risk Factor–Region Interactions for Asthma and GERD

Negative Binomial Regression of Asthma Risk Factor–Region Interactions					
**Exposure**	**Region**	**RR**	**Lower**	**Upper**	**LR_P_interaction**
Ambient Ozone Pollution	East Asia	0.864	0.807	0.925	0.00012
Ambient Ozone Pollution	Tropical Latin America	1.073	0.922	1.248	0.00012
Ambient Ozone Pollution	High-income North America	1.015	0.933	1.104	0.00012
Suboptimal Breastfeeding	East Asia	1.204	0.860	1.686	0.00020
Suboptimal Breastfeeding	Tropical Latin America	1.904	1.591	2.278	0.00020
Suboptimal Breastfeeding	High-income North America	1.226	1.011	1.487	0.00020
High Fasting Plasma Glucose	East Asia	1.231	1.179	1.285	0.00000
High Fasting Plasma Glucose	Tropical Latin America	1.163	1.136	1.192	0.00000
High Fasting Plasma Glucose	High-income North America	0.969	0.948	0.991	0.00000
High Body Mass Index	East Asia	3.944	2.834	5.490	0.00000
High Body Mass Index	Tropical Latin America	1.540	1.234	1.922	0.00000
High Body Mass Index	High-income North America	1.993	1.601	2.482	0.00000
Diet Low in Vegetables	East Asia	0.710	0.482	1.045	0.00000
Diet Low in Vegetables	Tropical Latin America	1.604	1.327	1.939	0.00000
Diet Low in Vegetables	High-income North America	1.000	0.620	1.615	0.00000
Diet High in Red Meat	East Asia	1.105	0.990	1.234	0.00001
Diet High in Red Meat	Tropical Latin America	0.817	0.742	0.899	0.00001
Diet High in Red Meat	High-income North America	1.224	0.901	1.663	0.00001
Diet High in Processed Meat	East Asia	43.040	21.820	84.897	0.00000
Diet High in Processed Meat	Tropical Latin America	16.602	5.962	46.234	0.00000
Diet High in Processed Meat	High-income North America	1.964	1.664	2.319	0.00000
Diet High in Sugar Sweetened Beverages	East Asia	2.163	1.389	3.367	0.00000
Diet High in Sugar Sweetened Beverages	Tropical Latin America	1.141	0.886	1.470	0.00000
Diet High in Sugar Sweetened Beverages	High-income North America	1.246	0.993	1.564	0.00000
Low Physical Activity	East Asia	1.395	1.179	1.652	0.00000
Low Physical Activity	Tropical Latin America	1.112	0.949	1.303	0.00000
Low Physical Activity	High-income North America	1.211	0.999	1.468	0.00000
High Temperature	East Asia	1.086	1.008	1.171	0.00080
High Temperature	Tropical Latin America	0.817	0.695	0.961	0.00080
High Temperature	High-income North America	1.008	0.977	1.040	0.00080
Low Temperature	East Asia	0.926	0.638	1.343	0.02107
Low Temperature	Tropical Latin America	1.603	1.245	2.064	0.02107
Low Temperature	High-income North America	1.027	0.924	1.140	0.02107
**Negative Binomial Regression of Gastroesophageal Reflux Disease(GERD) Risk Factor–Region Interactions**					
**Exposure**	**Region**	**RR**	**Lower**	**Upper**	**LR_P_interaction**
Ambient Ozone Pollution	East Asia	0.982	0.966	0.999	0.00000
Ambient Ozone Pollution	Tropical Latin America	1.009	0.988	1.031	0.00000
Ambient Ozone Pollution	High-income North America	1.208	1.148	1.272	0.00000
Suboptimal Breastfeeding	East Asia	1.079	1.019	1.143	0.00000
Suboptimal Breastfeeding	Tropical Latin America	1.040	1.005	1.076	0.00000
Suboptimal Breastfeeding	High-income North America	1.653	1.567	1.744	0.00000
High Fasting Plasma Glucose	East Asia	1.008	0.992	1.024	0.00000
High Fasting Plasma Glucose	Tropical Latin America	0.999	0.995	1.004	0.00000
High Fasting Plasma Glucose	High-income North America	0.931	0.919	0.944	0.00000
High Body Mass Index	East Asia	0.965	0.805	1.157	0.00000
High Body Mass Index	Tropical Latin America	0.964	0.890	1.044	0.00000
High Body Mass Index	High-income North America	0.710	0.634	0.795	0.00000
Diet Low in Vegetables	East Asia	0.943	0.889	1.000	0.00000
Diet Low in Vegetables	Tropical Latin America	0.993	0.977	1.009	0.00000
Diet Low in Vegetables	High-income North America	0.314	0.274	0.360	0.00000
Diet High in Red Meat	East Asia	1.044	1.016	1.072	0.00000
Diet High in Red Meat	Tropical Latin America	1.056	1.021	1.092	0.00000
Diet High in Red Meat	High-income North America	0.621	0.552	0.698	0.00000
Diet High in Processed Meat	East Asia	0.829	0.648	1.061	0.33620
Diet High in Processed Meat	Tropical Latin America	0.737	0.597	0.912	0.33620
Diet High in Processed Meat	High-income North America	0.669	0.629	0.710	0.33620
Diet High in Sugar Sweetened Beverages	East Asia	1.063	0.934	1.209	0.00000
Diet High in Sugar Sweetened Beverages	Tropical Latin America	1.014	0.939	1.095	0.00000
Diet High in Sugar Sweetened Beverages	High-income North America	0.796	0.752	0.844	0.00000
Low Physical Activity	East Asia	1.264	1.186	1.348	0.00000
Low Physical Activity	Tropical Latin America	1.183	1.124	1.246	0.00000
Low Physical Activity	High-income North America	1.087	0.993	1.190	0.00000
High Temperature	East Asia	1.040	1.016	1.064	0.00016
High Temperature	Tropical Latin America	1.064	1.012	1.118	0.00016
High Temperature	High-income North America	0.942	0.895	0.991	0.00016
Low Temperature	East Asia	0.802	0.714	0.901	0.06562
Low Temperature	Tropical Latin America	0.875	0.794	0.964	0.06562
Low Temperature	High-income North America	1.040	0.854	1.265	0.06562

References

1. Zhang D, Liu S, Li Z, Wang R. Global, regional and national burden of gastroesophageal reflux disease, 1990-2019: update from the GBD 2019 study. *Ann Med* 2022; **54**(1): 1372-84.
2. Li N, Yang WL, Cai MH, et al. Burden of gastroesophageal reflux disease in 204 countries and territories, 1990-2019: a systematic analysis for the Global Burden of disease study 2019. *BMC Public Health* 2023; **23**(1): 582.
